# Supplementary material for: National trends and resource associated with recurrent penetrating injury
Source: PLoS One. 2023 Nov 15;18(11):e0280702. doi: 10.1371/journal.pone.0280702 (PMC10650986; doi:10.1371/journal.pone.0280702)
Supplement: S1 Table — (DOCX) [file pone.0280702.s001.docx]

| Characteristics | ECodes | ICD-10-CM codes |
| --- | --- | --- |
| Gunshot Injury | E965x, E922x, E955x, E970,E9794, E985x | X940XXA, X941XXA, X942XXA, X948XXA, X949XXA, X9501XA, X9502XA, X9509XA, X958XXA, X959XXA, X93XXXA, W320XXA, W321XXA, W3301XA, W3300XA, W3302XA, W3303XA, W3309XA, W3310XA, W3311XA, W3312XA, W3313XA, W3319XA, W3400XA, W34010A, W34011A, W34018A, W3409XA, W3410XA, W34110A, W34111A, W34118A, W3419XA, X72XXXA, X730XXA, X731XXA, X732XXA, X739XXA, X738XXA, X7401XA, X7402XA, X7409XA, X748XXA, X749XXA, Y35003A, Y35002A, Y35009A, Y35011A, Y35012A, Y35013A, Y35019A, Y35021A, Y35022A, Y35023A, Y35029A, Y35031A, Y35032A, Y35033A, Y35039A, Y35041A, Y35042A, Y35043A, Y35049A, Y35091A, Y35092A, Y35093A, Y35099A, Y22XXXA, Y230XXA, Y231XXA, Y232XXA, Y233XXA, Y238XXA, Y239XXA, Y240XXA, Y248XXA, Y249XXA |
| Stab Injury | E919x, E966, E974, E956, E920x, E919x | X990XXA, X991XXA, X992XXA, X998XXA, X999XXA, Y35401A, Y35402A, Y35403A, Y35409A, Y35411A, Y35412A, Y35413A, Y35419A, Y35491A, Y35492A, Y35493A, Y35499A, W240XXA, W241XXA, W25XXXA, W260XXA, W261XXA, W262XXA, W268XXA, W269XXA, W28XXXA, W293XXA, W292XXA, W294XXA, W291XXA, W298XXA, W300XXA, W301XXA, W302XXA, W303XXA, W3081XA, W3089XA, W309XXA, W310XXA, W311XXA, W312XXA, W313XXA, W3181XA, W3182XA, W3183XA, W3189XA, W319XXA, X780XXA, X781XXA, X782XXA, X788XXA, X789XXA |

**Supplemental Table S1**. International Classification of Disease, Ninth and Tenth revision, Codes (ICD-9/10) for Penetrating Traumas.
