# Supplementary material for: National trends and resource associated with recurrent penetrating injury
Source: PLoS One. 2023 Nov 15;18(11):e0280702. doi: 10.1371/journal.pone.0280702 (PMC10650986; doi:10.1371/journal.pone.0280702)
Supplement: Attachment — Submitted filename: Point-by-Point_Cover Letter.doc [file pone.0280702.s002.doc]

**David Geffen School of Medicine at UCLA**

**Ronald Reagan-UCLA Medical Center**

Department of Surgery

Division of Cardiac Surgery

David Geffen School of Medicine at UCLA

62-249 Center for Health Sciences

Los Angeles, CA 90095-1741

Tel: 310-206-6717

Email: PBenharash@mednet.ucla.edu

PEYMAN BENHARASH, MD

Associate Professor of Surgery and Bioengineering

Associate Director Thoracic Residency Program

Director of Adult ECMO Program at UCLA

Director of UCLA Cardiac Outcomes Research Laboratories

June 7, 2023

**RE: Revisions for manuscript entitled “National Trends and Resource Utilization Associated with Recurrent Penetrating Injury”**

Dear Dr. Chenette and the esteemed members of the Editorial Board,

On behalf of my co-authors, I would like to thank the reviewers for their thorough review of our manuscript. We deeply appreciate the profound and nuanced comments made by the reviewers and hope that we have adequately addressed them. Please find our point-by-point responses below. The pertinent modifications are highlighted in the revised manuscript.

We express our sincerest appreciation to the editorial team and reviewers for the providing us the opportunity to revise the manuscript titled above for potential publication in *PLOS One*. We look forward to your team’s thorough review of our manuscript and would be happy to provide any further information as deemed necessary. Thank you for your invaluable time and your thoughtful consideration.

Sincerely,

Peyman Benharash, MD

**GENERAL COMMENTS:**

**- First, although the term “trauma recidivism” is common, I would caution the authors against its use. The term “recidivism” is strongly associated with criminality and implies fault on the part of the patient. Patients injured due to violence frequently come from marginalized groups, and this language risks reinforcing racist assumptions about this patient population. Recurrent violent injury is a clear phrase that can be substituted. I recommend authors to use “recurrent traumatic injury” instead.**

*We thank you for this comment. Following the editor’s recommendation, the term “trauma recidivism” has been replaced with recurrent penetrating injury (RPI) throughout the manuscript.*

**- Please follow the STROBE guidelines style, especially for the methods section**

**ABSTRACT:**

**- Results: please provide numerical results**

*Thank you for this point. We have updated results section of the abstract with numerical results.*

**INTRODUCTION:**

**- Introduction, line 1: What does “urban trauma” mean? Is this study limited to urban areas?**

**- I recommend that the aim of the study be explicitly stated at the end of the introduction.**

*We appreciate this comment. The word “urban trauma” has been replaced with “traumatic injuries” to minimize confusions, as this study was not limited to urban areas. Last two sentences of the Introduction has been revised per recommendation: “Thus, the present study examined national trends in RPI as well as factors associated with it. The aim of this study was to elucidate clinical and financial outcomes following RPI using a nationally representative cohort” (Page 4).*

**METHODS**

**- How were new injuries distinguished from patients readmitted due to sequelae or complications of their index admission?**

**- How was the index admission identified? Could patients have been injured prior to the first visit that is denoted here?**

**- The study uses ICD-9/10 codes to identify firearm- and stab-related trauma, which may not capture all cases or accurately differentiate between intentional and unintentional injuries. How did authors handle this?**

**- Proportion of missing data, figure 1, supplemental Table 1 are results and should not be mentioned in the methods section but in the results section instead.**

*We thank you for your valuable suggestions. Although ICD-10 codes differentiates between initial, subsequent and sequelae of given diagnosis, we are unable to clearly delineate whether patients were readmitted due to sequelae or complications of their index admission. We have noted the limitation as follows: “Due to our reliance on ICD-10 diagnosis and coding practices at various institutions to define penetrating injuries, patients readmitted due to sequelae or complications of their index admissions could not be clearly delineated” (Page 12).*

*As noted on our Supplemental Table S1, only codes for initial encounter of injuries were used to determine index admission.*

*Both the External Cause of Injury and International Classification of Diseases, Tenth Revision codes have enough granularities to show whether the injuries were intentional or non-intentional. Intentions of the penetrating injuries are noted on Table 2 and Supplemental Table S2.*

*Proportion of missing data, figure 1 and supplemental Table 1 have been removed from the Method section as recommended.*

**RESULTS**

**- Figure 1: please indicate how many patients were excluded for each exclusion criteria.**

**- Please provide n and % in tables not only %**

**- Please indicate the evolution of % GSW between 2010 and 2019**

**- Paragraph 3.3: Of an estimated 687,863 patients, 15,142 (2.2%) were (and not was) identified.**

*Thank you for this comment on the result section. We have revised the Figure 1 and Table 1 as advised. Evolution of recurrent GSW as well as percent increase in recurrent GSW hospitalizations were noted in the Result section (Page 7). Paragraph 3.3 was edited as recommended.*

**DISCUSSION**

**- Discussion, line 1: “Penetrating trauma is the strongest predictor of future traumatic injury.” This is an often-repeated saying, but to my knowledge it has not been demonstrated. The cited reference here simply refers to Sims’ 1989 paper, which does not establish this relationship.**

*We appreciate your insightful point. First sentence of the discussion has been modified as the following: “Penetrating trauma has been associated with recurrence and is responsible for >$25 billion in annual costs based on medical expenses and lost productivity in the US.” (Page 9).*

**- By using the NRD, this analysis is limited to injuries that result in hospitalization. This is less than half of firearm injuries, with the majority being treated and released from the ED. If this data source is to be used, all of the results and conclusions must be interpreted in this context.**

*We thank you for this comment. We have updated the definition of our cohort as the following in the Method section: “Recurrent Penetrating Injury was defined as a subsequent penetrating injury requiring readmission within 60 days of discharge from the index hospitalization.” (Page 5). Furthermore, we have updated our result section to emphasize that our cohort definition of hospitalization due to recurrent penetrating injury with following sentences below:*

*“From 2010 to 2019, hospitalizations due to gunshot wound injury increased by 138% and recurrent GSW by 196% in annual incidence (nptrend<0.001, Figure 2).” (Page 7).*

*“Over the study period, there was a stable trend in hospitalization due to recurrent stab injury (nptrend=0.60, Figure 2).” (Page 8).*

*Furthermore, in order to emphasize our limitation regarding those treated and released from the ED, we added the following sentence to the limitation section: “Despite the strength of NRD to track repeat hospitalizations at participating hospitals, information regarding the outpatient treatment of RPI as well as out-of-hospital mortality are unable to be determined.”*

**- I believe the NRD does not include race. Given the role of structural racism in generating risk for violent injury, this is a limitation that should be mentioned.**

*Thank you for this comment. The limitation section of the discussion has been edited with the following statement: “Additionally, the NRD lacks patient-identifying variables, such as race, location, and hospital data, as well as surgeon-specific variables.” (Page 12).*

**- 30% of readmissions were very soon after discharge. Again, this makes me suspect that these are either transfers or complications, rather than real new injuries. Please clarify this point**

*Thank you for this point. Indeed, days to readmissions have been reported to be very soon after discharge since our analysis of timing was limited by the database. As noted in our Method section, the NRD tracks readmissions “within each calendar year.” Readmissions across the years were unascertainable. As we acknowledge these timing to readmissions could be misleading, we have removed them from our results section. Additionally, as we rely on ICD coding for our definition of recurrent penetrating injury cohort, our limitation section has been updated accordingly to account for any possible misattribution.*

**- Patients discharged to rehab, etc, had higher risk of readmission. Is it really plausible that these were new injuries? Patients with injuries that are severe enough to require rehab are less likely to be out and about. And indeed, since much of the 60 day follow up period would be spent in rehab, I suspect this may be misattribution.**

*We appreciate your comment. As shown in Table 3, for those discharged to skilled nursing facilities and home health, only those with GSW have been shown to have a higher risk of readmission within 60 days. This may be due to the misattribution from patients undergoing sequelae events or complications, and this has now been noted in the limitations of our manuscript. Nonetheless, our data showed that, among those hospitalized for both recurrent gunshot and stab injuries, transfer to a short-term care facility and leaving against medical advice are associated with an increased risk of readmission. Leaving against medical advice and transferring to a short-term medical facility may limit appropriate follow-up care and contribute to new injuries.*

**- The conclusion of the study should be appropriately restrained and aligned with its objective**

*Thank you for this comment. We have revised our conclusion as the following: “The rate of RPI among patients with penetrating trauma has increased over the past decade and continues to pose a clinical and financial burden in the US. Our study underscores the contributing factors underlying repetitive incidences of gunshot and stab injuries. In light of these findings, national efforts to improve post-discharge preventive measures and bolster social support services for patients with penetrating trauma are warranted. To effectively address the mounting burden of RPI, multidisciplinary approaches involving physicians, public health leaders, and legislators should be adopted to mitigate the incidence of penetrating trauma within our communities.” (Page 12-13).*
